# The human "magnesome": detecting magnesium binding sites on human proteins

**DOI:** 10.1186/1471-2105-13-S14-S10

**Published:** 2012-09-07

**Authors:** Damiano Piovesan, Giuseppe Profiti, Pier Luigi Martelli, Rita Casadio

**Affiliations:** 1 Biocomputing Group, Department of Biology, University of Bologna, Bologna, 40126, Italy; 2 Health Science and Technologies-ICIR, University of Bologna, Bologna, 40126, Italy

## Abstract

**Background:**

Magnesium research is increasing in molecular medicine due to the relevance of this ion in several important biological processes and associated molecular pathogeneses. It is still difficult to predict from the protein covalent structure whether a human chain is or not involved in magnesium binding. This is mainly due to little information on the structural characteristics of magnesium binding sites in proteins and protein complexes. Magnesium binding features, differently from those of other divalent cations such as calcium and zinc, are elusive. Here we address a question that is relevant in protein annotation: how many human proteins can bind Mg^2+^? Our analysis is performed taking advantage of the recently implemented Bologna Annotation Resource (BAR-PLUS), a non hierarchical clustering method that relies on the pair wise sequence comparison of about 14 millions proteins from over 300.000 species and their grouping into clusters where annotation can safely be inherited after statistical validation.

**Results:**

After cluster assignment of the latest version of the human proteome, the total number of human proteins for which we can assign putative Mg binding sites is 3,751. Among these proteins, 2,688 inherit annotation directly from human templates and 1,063 inherit annotation from templates of other organisms. Protein structures are highly conserved inside a given cluster. Transfer of structural properties is possible after alignment of a given sequence with the protein structures that characterise a given cluster as obtained with a Hidden Markov Model (HMM) based procedure. Interestingly a set of 370 human sequences inherit Mg^2+ ^binding sites from templates sharing less than 30% sequence identity with the template.

**Conclusion:**

We describe and deliver the "human magnesome", a set of proteins of the human proteome that inherit putative binding of magnesium ions. With our BAR-hMG, 251 clusters including 1,341 magnesium binding protein structures corresponding to 387 sequences are sufficient to annotate some 13,689 residues in 3,751 human sequences as "magnesium binding". Protein structures act therefore as three dimensional seeds for structural and functional annotation of human sequences. The data base collects specifically all the human proteins that can be annotated according to our procedure as "magnesium binding", the corresponding structures and BAR+ clusters from where they derive the annotation (http://bar.biocomp.unibo.it/mg).

## Background

Magnesium is the most abundant divalent alkaline ion in living cells and it is an indispensable element for many biological processes. Magnesium deficiency in humans is responsible for many diseases including osteoporosis [1] or metabolic syndrome (MetS), a combination of different metabolic disorders that increase the risk of developing cardiovascular diseases and diabetes [2]. Magnesium is characterised by specific chemico-physical properties: it is redox inert, it has a small ionic radius and is consequently endowed with a high charge density [3,4]. In cells magnesium ions have both structural and functional roles. Magnesium plays a key role in stabilising protein structures, phosphate groups of membrane lipids and negatively charged phosphates of nucleic acids. Concomitantly, it is also involved in catalytic roles, such as the activation/inhibition of many enzymes [3,4].

Observations on the structural geometry of Mg^2+ ^binding sites in proteins known with atomic resolution may be derived from PROCOGNATE, a cognate ligand domain mapping for enzymes [5] and from the Protein Data Bank [PDB, http://www.rcsb.org]. Typical magnesium binding sites on proteins show three or fewer direct binding contacts with carbonyl oxygen atoms of the backbone and/or protein side chains, with a tendency to bind water molecules given the octahedral coordination geometry of the divalent cation [3,6]. It is known that Mg^2+ ^binding sites are less specific than those of other divalent cations such as Zn^2+ ^and Ca^2+^, and that in particular conditions, Zn^2+ ^can dislocate Mg^2+ ^from its pocket [3,7]. Apparently metal binding sites on proteins seem to satisfy constraints related to the physiological availability of the ions [4]. Magnesium binds weakly to proteins and enzymes (Ka≤ 10^5 ^M^-1^) [8] and its binding affinity appears to be dependent on its high cellular concentration. Free Mg^2+ ^concentration is higher than that of any other ion (0.5-1mM, [4]). As a consequence magnesium binding sites are less conserved through evolution than those of others divalent cations [4] and their detection is therefore difficult. Mg^2+ ^binding sequence motifs have been described to be conserved in similar RNA and DNA polymerases [9,10]. Three dimensional Mg^2+ ^binding pockets derived from 70 Mg^2+ ^binding proteins solved at atomic resolution were recognised in protein structures by implementing a structural alphabet [11].

In this work we describe how to assign putative Mg^2+ ^binding sites to human proteins that lack structural information and also to proteins that share less than 30% sequence identity with any available Mg^2+ ^binding protein template. This is possible within our BAR-PLUS annotation resource (BAR+), a non hierarchical clustering method that has been recently described and relies on the pair wise sequence comparison of about 14 millions proteins, including 998 complete proteomes of different species and *Homo sapiens *[12,13]. This paper to our knowledge describes the first large scale investigation of magnesium binding sites at the human proteome level. The results highlight that residues involved in magnesium binding in protein structures (derived from the PDB) falling into the same BAR+ cluster are conserved and can be transferred to all the human sequences sharing the same cluster on the basis of structure to sequence alignment with a cluster specific hidden Markov model (HMM). Magnesium binding sites within a given cluster are also conserved when pair-wise sequence identity among the target and the template/s is less than 30%. A data base (BAR-hMG) is made available from where for a given human input sequence the predicted magnesium binding site/s can be retrieved with the corresponding structural template/s and the annotating BAR+ cluster.

## Methods

### The dataset of Mg^2+ ^binding protein structures

A list of 4,710 magnesium binding protein structures was retrieved from the Ligand-Expo database [14] by searching "MG" as Mg^2+ ^ligand identifier. The Expo database is a data warehouse that integrates databases, services and tools related to small molecules bound to macromolecules and based on PDB. It allows users to extract ligand information directly from the PDB, to perform chemical substructure searches of PDB ligands using a graphical interface and also to browse other relevant small molecule resources on the Web. It is updated daily and therefore provides the most current information on small molecules present in the PDB. Its reliability is based on the reliability of the structures from where information is derived and ultimately on the resolution of the electron density map of the molecule. Our set includes PDBs with an average Resolution (R) factor of 0.23 nm. The list of magnesium binding residues and corresponding positions in the sequence for each PDB was obtained parsing both the "LINK" and "SITE" fields on the coordinate files [15]. In order to guarantee that magnesium is part of a biologically significant PDB structure, we filtered out fragments and chimeric structures by constraining the coverage of the template PDB structure to its UniProtKB corresponding sequence (without signal peptide, when present) to be ≥70%. This bound guarantees a satisfactory overlapping of the sequence to its structure and this is essential in building by homology procedures. Applying this criterion, we ended up with 1,341 PDB templates. For each PDB structure the reference sequence and the corresponding UniProtKB [16] accession are obtained from the Sifts web server [17]. In case of multiple PDBs containing different magnesium binding sites and referring to the same sequence, all the sites are mapped into the protein sequence. Human sequences are collected from UniProtKB (release 2011_02), including also splicing isoforms, for a total of 110,464 sequences. Most of these sequences are annotated in UniProtKB in an automatic way and lack any experimental evidence. When fragments are filtered out, the total number of human sequences adopted for our analysis is 84,520.

### The BAR-PLUS annotation resource

BAR+ is an annotation resource based on the notion that sequences with high identity value to a counterpart can inherit from this the same function/s and structure, if available (http://bar.biocomp.unibo.it/bar2.0/). The method has been recently described [13]. Briefly, an extensive BLAST alignment [18] was performed for some 13,495,736 sequences in a GRID environment [13]. The sequence similarity network was built by connecting two proteins only if their sequence identity is ≥40% with an overlap (Coverage, COV) ≥90%. 913,762 clusters were obtained by splitting of the connected components of the similarity network. Mapping of PDB, Pfam functional domains (http://pfam.sanger.ac.uk/) and GO terms (Gene Ontology terms, http://www.geneontology.org/) as listed in the UniProtKB protein files allows different annotation types within each cluster. Enrichment of Pfam domains [http://www.sanger.ac.uk/resources/databases/pfam.html] and GO terms [http://www.geneontology.org/] for each cluster was statistically validated (by computing a Bonferroni corrected P-value and by selecting its significance threshold with a bootstrapping procedure) [13]. Only when P<0.01, terms are transferred from one protein to another one in the same cluster and annotation is inherited by all the sequences in the cluster. When a sequence falls into a validated cluster it can inherit in a validated manner functional and structural annotation (PDB +/SCOP +/Pfam +/GOterms +/). Stand alone sequences are called Singletons (30.4% of the total protein universe). Clusters can contain distantly related proteins that by this procedure can be annotated with high confidence. We verified that the magnesium containing 1,341 PDB structures were in BAR+ clusters and when not present, we included them in the corresponding cluster. In any case we verified that backbone structure was conserved in the same cluster (average Root Mean Square Deviation (RMSD) was about 2.0±0.2 Å) (for the definition of RSMD see: http://cnx.org/content/m11608/latest/). The human sequences were then aligned against BAR+ clusters and only those satisfying the BAR+ constraints (ID≥40% and COV≥90%) were retained. Out of the 84,520 human sequences aligned towards BAR+ with the required criteria, some 61,106 fell into 22,858 clusters and some 2,791 aligned with singletons. The remaining portion of the human proteome (aligned with sequences contained in BAR+ clusters with lower sequence identity and coverage than those required for a validated transfer of annotation) is not considered in the present analysis. In BAR+, each cluster endowed with structure/s is characterised by a computed cluster Hidden Markov Model (HMM) that is derived from a structure-to-sequence alignment within the cluster and can be adopted to model the cluster sequences on the structure template/s of the cluster [12]. We took advantage of the cluster HMM both for structural alignments of the newly introduced PDB structures and for sequence-to-structure alignment.

### Selection of the "human magnesome"

Out of the above selected 61,106 human sequences, we focused on the subset that comprises all the chains included in 251 clusters endowed with magnesium containing PDB structures. In our clusters, we deal with 1,341 PDBs. We therefore checked all the PDB files, the corresponding UniProtKB files and the related literature. From this effort we were able to verify that for only 119 structures (9% of the total) in 21 clusters there is no published observation supporting so far any functional or structural role of MG. Within the clusters, sequences could also safely inherit validated Pfam functional domains and GO functional terms (Molecular Function, Biological Process and Cellular Component, http://www.geneontology.org/).

Binding positions were transferred from the template/s to the target after pair-wise alignment/s based on the cluster HMM. 251 clusters contain Mg binding templates and there from an equivalent number of HMM models were used to transfer Mg binding position/s to the human sequences in the clusters. 141 clusters contain 827 magnesium binding protein structures derived from non human species (25 different Eukaryota, 42 different bacteria, 9 different Archaea and 1 virus). 110 clusters contain 514 human templates.

## Results and discussion

### Finding Magnesium binding sites with BAR+

**Figure 1:**
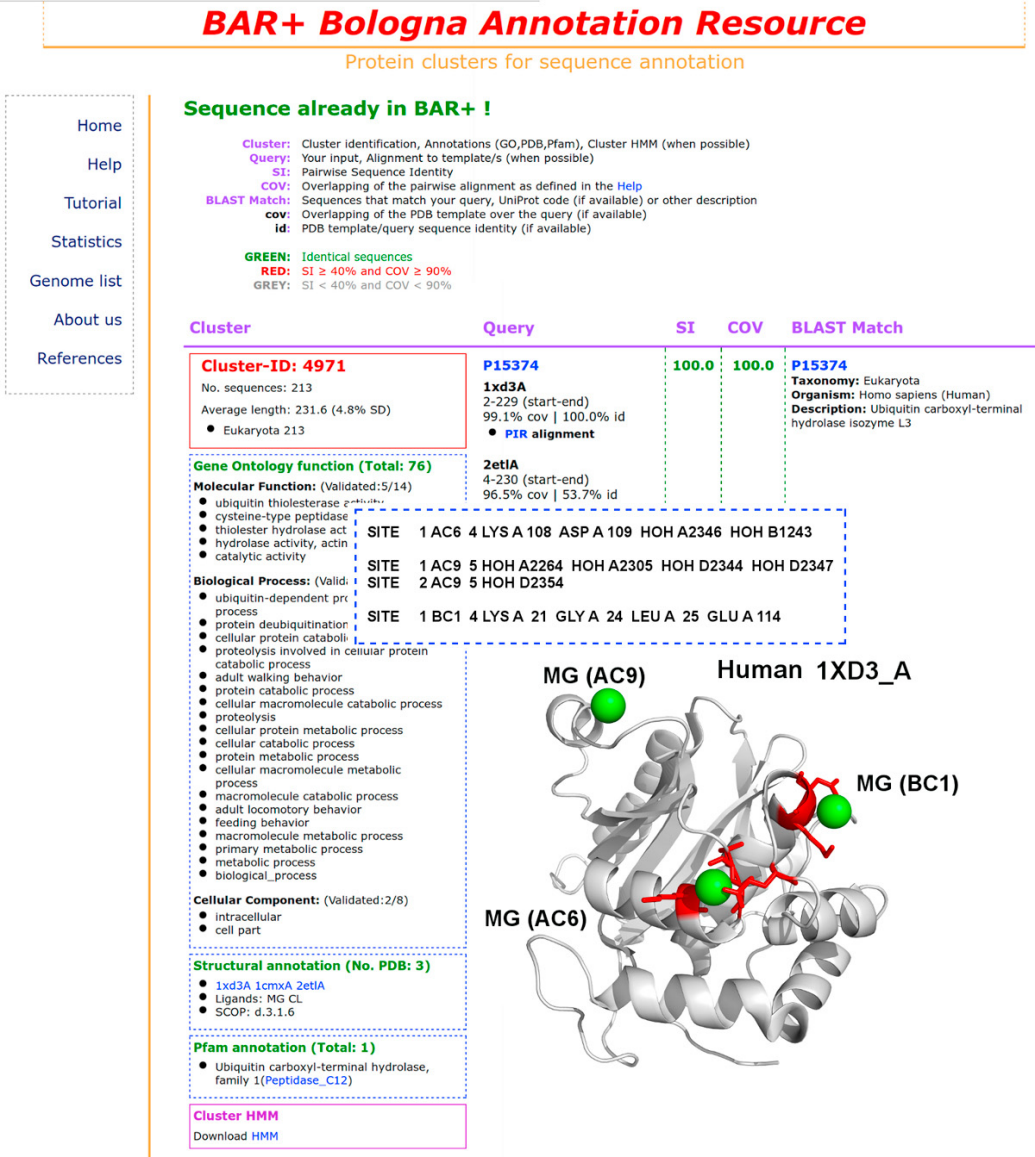
**A BAR+ cluster with a magnesium binding template**. The BAR+ output. When the query is the UniProtKB accession code P15374, the corresponding annotation cluster comprises 213 sequences from Eukaryotes with an average length of 232 residues and 3 PDB structures. Only one of them (human Ubiquitin hydrolase UCH-L3, PDB:1XD3_A) is shown using PyMol (http://www.pymol.org) with the three Mg ions. Of the three ions (as shown in the inset where the PDB SITE fields are reported) only two are coordinated by lateral side chains (in red in the protein structure representation). The cluster contains 26 validated GO terms and 1 validated Pfam term (PF01088, Ubiquitin carboxyl-terminal hydrolase, family 1) that are also inherited by the human query sequence. See text for details.

When a human sequence has a counterpart in BAR+ with sequence identity ≥ 40% over at least 90% of the alignment length, it falls into the same cluster of the similar chain. In the example of Figure 1, when human sequence P09936 is aligned towards the BAR+ data base, the result web page identifies cluster #4791 that comprises 213 sequences from Eukaryotes with an average length of 232 residues (Standard Deviation (SD)=4.8%) and 3 PDB structures with magnesium and chloride ions as ligands (1CMX_A from *Saccharomyces cerivisiae*; 2ETL_A and 1XD3_A from *Homo sapiens*). The three templates are however highly similar (the average root mean square deviation is 1.62+/-0.35Å). Here we focus only on magnesium binding sites and for clarity we show only the structure of the human Ubiquitin hydrolase UCH-L3 (1XD3_A). As shown, the structure contains 3 Mg ions. The Site field of the corresponding PDB file indicates that of the three Magnesium ions one is coordinated only by water molecules and it is not considered in our analysis. The remaining two are coordinated by four and two residues, respectively (the remaining coordination sites are probably occupied by water). With the cluster HMM based alignment only the coordination sites including residues of the template/s are transferred to the human sequences falling into the cluster. From the cluster, the human sequence inherited all the validated features that are reported in the corresponding web page: validated GO terms, the SCOP classification, and the Pfam domain PF01088 (Ubiquitin carboxyl-terminal hydrolase, family 1). BAR+ gives the HMM based target/template alignment for computational modelling of the 3D structure of all the other sequences in the cluster. Among these, 4 are from *Homo sapiens *and inherit all the cluster specific annotation, including the Mg binding sites.

Bound Mg in this structure is not as yet supported by any experimental observation highlighting a specific functional role. The whole BAR-hMG data base contains 21 out of 251 clusters with templates binding Mg without any experimental (still) determined functional or structural role. This information can be retrieved for each template from the corresponding PDB and UniProtKB files and the quoted literature therein. It should be considered that Mg ions may play a role on protein stability still not fully described or even a role in protein-protein interaction that is at the basis of many relevant biological processes. In many instances the formation of protein complexes has not yet been recognized due to its transient characteristics. Therefore the question is still open and we therefore included also these cases in our data set for a comprehensive analysis of putative Mg binding sites. Clusters containing templates where Mg has a documented structural and functional role are labelled with a yellow star, and a yellow star and the corresponding EC number, respectively. For this reason no label is present in the figure.

### Annotation of Mg^2+ ^binding sites in human proteins

A structural analysis of the magnesium containing 1,341 PDB templates indicates that the ion can be present in different ways. For this reason we list our annotation results considering that the ion co-crystallises with the protein chain either alone (Mg) or concomitantly with other ions (Mg and Ions) or ligands (Mg and Ligands) or with other ions and ligands (MG, Ions and Ligands). In some instances PDB structures can combine two or more of the binding modes (Mixed). Results are listed by splitting human sequences that inherited annotation from human templates (2,688) from those that inherit annotation from structures of other organisms (1,063). The results are shown in Table 1 and 2, respectively, where the number of sequences with low sequence identity to the cluster templates is also reported. Clusters are split depending on the role of bound Mg ion: functional, structural, not yet determined.

**Table 1:** Human sequences annotated with human structural templates

	Cluster (#)			PDB (#)	Cluster RMSD (Å)	Template sequence (#)	Annotated sequence (#)	Newly annotated sequence (#)	Annotated sequence (ID<30%)*
	*$*	*^*	*°*						
Mg	8	1	0	9	-	9	55	54	1
Mg and Ions	7	1	0	9	0.30	8	53	52	6
Mg and Ligands	24	4	2	73	0.77	32	159	158	33
Mg , Ions and Ligands	22	5	4	57	0.52	31	1948	1947	19
Mixed	22	6	4	366	0.68	92	473	455	120
Total	83	17	10	514		172	2688	2666	179

Human sequences that inherit annotation from human structural templates are listed as a function of the different typologies of magnesium binding in the PDB files. The table lists the number of clusters, of structural templates, of annotated sequences (sequences that inherit Mg binding positions) according to our procedure, of sequences never annotated before as Mg binding proteins according to UniProtKB and of *sequences annotated when the target/template identity is below the 30%. Three different types of clusters are identified and listed in the first column: $ cluster with structures binding MG with a recognized functional role and whit an EC number, ^ clusters with structures binding MG with a recognized structural role (without an EC number), ° cluster containing structures (119 out of 1,341) binding MG without recognized physiological role.

**Table 2:** Human sequences annotated with structural templates from other organisms

	Cluster (#)			PDB (#)	Cluster RMSD (Å)	Template sequence (#)	Annotated sequence (#)	Newly annotated sequence (#)	Annotated sequence (ID<30%)*
	*$*	*^*	*°*						
Mg	12	10	0	75	0.73	33	105	105	24
Mg and Ions	5	5	0	160	0.38	10	51	50	22
Mg and Ligands	20	22	3	81	0.86	54	359	352	51
Mg , Ions and Ligands	12	6	2	66	0.52	23	278	276	28
Mixed	21	17	6	445	0.83	95	270	243	66
Total	70	60	11	827		215	1063	1026	191

Table legend is as in Table 1.

The number of PDB human protein structures with bound magnesium (514) univocally identifies 172 template sequences; within the BAR+ environment this number reaches 2,688 (Annotation inherited from human templates). Some other 1,063 human sequences inherit annotation within BAR+ clusters where the structural templates are from other organisms (Table 2) (Annotation inherited from other organisms).

When more PDB structures fall into the same cluster (Table 1 and 2) their RMSDs are very low (<1 Å) for all the groups. This indicates that the BAR+ clusters preserve the structural specificity. Therefore when a target sequence falls into a cluster characterised by Mg binding, the corresponding site annotation can be safely inherited. This is so also for very distantly related sequences (sequence identity <30%, last column) that are in the same cluster.

In BAR-hMG some 3,751 human sequences are annotated as Mg binding. About 98% of this set is annotated for the first time. For these sequences the corresponding UniProtKB entry neither has any information on Mg binding nor contains any GO term related to Mg binding.

**Figure 2:**
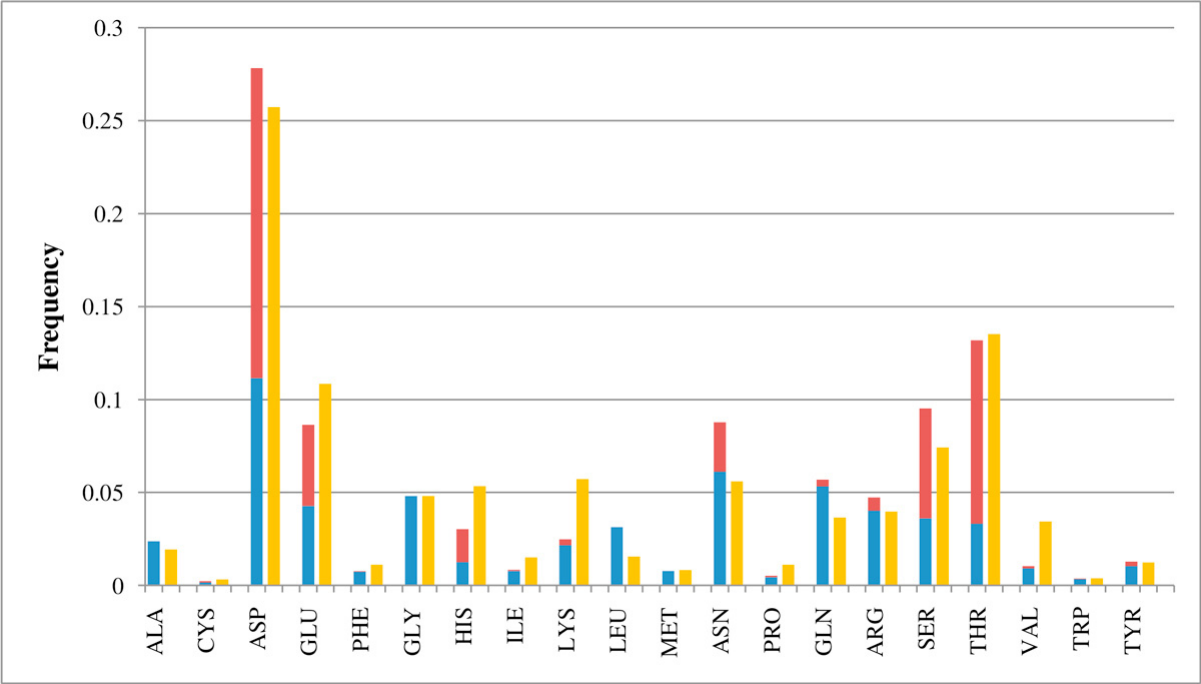
**Frequency distribution of Magnesium binding residues in PDB templates and in annotated human sequences**. Distribution of the frequency of residues coordinating magnesium ions in the PDB structures (1,341, blue color: Mg is coordinated by the backbone carbonyl oxygen, red color: Mg is coordinated by the lateral side chain) and in the putatively annotated human sequences (3,751, yellow color).

Characteristics of Mg^2+ ^binding sites can be detected from a simple counting on the retrieved 1,341 PDB structures contained in the 251 clusters of the BAR-hMG data base. Results (shown in Figure 2) are split into binding sites stabilised by lateral side chains and by backbone carbonyl groups. The highest frequency is observed for Asp and Glu residues. Similar frequency distribution is obtained when counting is done on the newly annotated human sequences (Figure 2). Here binding is referred only to the residue type.

### Localising the human Mg^2+ ^binding sequences

In Table 3 we list the most populated cellular localizations (Cellular Component of the Gene Ontology) of the human sequences (the "human magnesome") sorted out according to the different magnesium binding modes. For each GO term, the number of human sequences is reported. The selected terms are those that are the most distant from the ontology root in the corresponding BAR+ cluster of each sequence. Similarly GO terms of biological process and molecular function can be obtained for each sequence (data not shown; the data can be retrieved when a sequence falls into a validated cluster).

**Table 3:** Localising the human magnesium binding sequences

Sequence (#)	GO terms (Cellular Component)	Sequence (#)	GO terms (Cellular Component)
	**Mg**		**Mg + Ions + Ligands**
23	endoplasmic reticulum lumen	1817	cell surface
21	cell body	117	endoplasmic reticulum part
		
	**Mg + Ions**	92	dendrite cytoplasm
33	site of polarized growth	56	mitochondrial matrix
13	membrane-bounded organelle	48	cell division site
		
	**Mg + Ligands**	47	ruffle
118	azurophil granule	44	cell septum
37	cytoplasmic mRNA processing body	44	membrane raft
19	cytoplasmic membrane-bounded vesicle	37	endoplasmic reticulum
16	intracellular	24	cell leading edge
15	intracellular membrane-bounded organelle	23	plasma membrane enriched fraction
14	mitochondrion	22	internal side of plasma membrane
11	neuron projection	15	cell cortex
11	cell part	15	intracellular membrane-bounded organelle

For explanation see text.

### The "Human Magnesome" database

**Figure 3:**
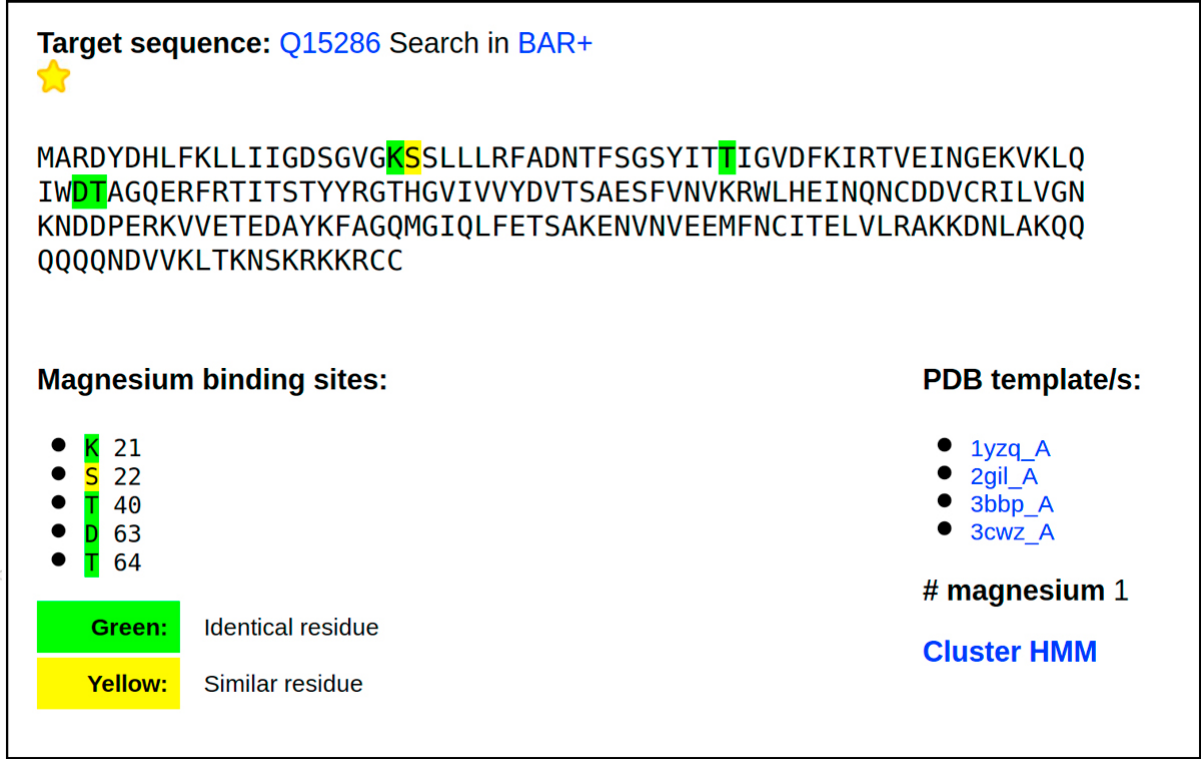
**The Human Magnesome output**. A typical output of the human magnesome site (BAR-hMG). The test sequence inherits, after cluster based HMM alignment to the corresponding templates (listed in the inset), five binding residues (K 21, S 22, T 40, D 63 and T 64). The residues are color coded depending on the BLOSUM 62 alignment scoring matrix. From the result page is also possible to retrieve the matching BAR+ cluster page and the corresponding UniProtKB page of the target entry. The green color in the output indicates residues identical to the original template/s. Similar residues are highlighted in yellow. The yellow star indicates that the protein is located in a cluster where Mg binds to PDB templates (listed) in a documented structural way. Cluster HMM can be downloaded.

The "Human Magnesome" is a data base of human sequences generated after annotation with the procedure here described. The main page allows a sequence search either with a UniprotKB accession code or the FASTA format of the sequence. When the sequence is present in the database it is returned with the putative magnesium binding sites, the structural templates from where it inherits magnesium binding and the number of magnesium ions present in the structural templates. Different colors are displayed when the binding residues are identical, similar or different to the template reference/s. Residue substitution is scored with Blosum62 matrix. In Figure 3 a typical output is shown. The data base is available at http://bar.biocomp.unibo.it/mg.

## Conclusion

In this work we address the problem of annotating magnesium binding sites in proteins starting from their sequence. We take advantage of an annotation resource recently introduced (BAR+, [13]), where functional and structural features derived from PDB structures are implemented into HMM models that allows sequence to template alignment even when sequence identity is below 30%. This procedure is based on the notion of "cluster", a set of sequences retrieved as connected components of a graph where two proteins are linked together when they share a sequence identity greater or equal than 40% in at least 90% of the pair wise alignment length. By restricting our analysis to clusters containing human sequences and magnesium binding PDB structures, we align with the cluster HMMs some 3,751 human sequences that fall in the same clusters and inherit by this the magnesium binding feature. Some 370 human sequences share an identity to the template less than 30%.

We therefore prove feasible that magnesium binding sites can be inherited from a given template when the sequence falls inside a well annotated cluster from where it derives also validated Pfam functional domains and GO functional terms. Presently we can annotate some 5% of the human genome as inheriting the capability of binding magnesium ions. All the analysed sequences, their binding sites, and the corresponding clusters from where they derive annotation are included in the Human Magnesome data set (BAR-hMG), freely available at http://bar.biocomp.unibo.it/mg.

## Competing interests

The authors declare that they have no competing interests.

## Authors' contributions

DP carried out all the calculations. GP developed the web site. RC, DP, GP and PM conceived the study, analyzed the data and wrote the manuscript. All the authors have read and approved the final manuscript.
